# Cerebral and Extracranial Neurodegeneration are Strongly Coupled in Parkinson’s Disease

**DOI:** 10.2174/1874205X00701010001

**Published:** 2007-08-22

**Authors:** Jörg Spiegel, Dirk Hellwig, Wolfgang H Jost, Georgios Farmakis, Samuel Samnick, Klaus Fassbender, Carl M Kirsch, Ulrich Dillmann

**Affiliations:** 1 Department of Neurology Saarland University, D-66421 Homburg/Saar, Germany; 2 Department of Nuclear Medicine, Saarland University, D-66421 Homburg/Saar, Germany; 3 Department of Neurology, Deutsche Klinik fur Diagnostik, D-65191 Wiesbaden, Germany

**Keywords:** Parkinson’s disease, FP-CIT SPECT, MIBG scintigraphy

## Abstract

In idiopathic Parkinson’s disease (PD), a generalized Lewy body type-degeneration in the brain as well as extracranial organs was identified. It is unclear, whether cerebral and extracranial Lewy body type-degeneration in PD are coupled or not. To address this question, cerebral [^123^I]FP-CIT SPECT – to quantify cerebral nigrostriatal dopaminergic degeneration – and myocardial [^123^I]MIBG scintigraphy – to quantify extracranial myocardial sympathetic degeneration – were performed in 95 PD patients and 20 healthy controls. At each Hoehn and Yahr stage separately, myocardial MIBG uptake correlated significantly with striatal FP-CIT uptake. No such correlation was found in the controls. Cerebral and extracranial Lewy body type-degeneration in PD do not develop independently from each other but develop in a strongly coupled manner. Obviously cerebral and extracranial changes are driven by at least similar pathomechanisms. Our findings in controls contradict a physiological correlation between nigrostriatal dopaminergic and myocardial sympathetic function.

## INTRODUCTION

Idiopathic Parkinson’s disease (PD) results from a Lewy body type-degeneration of dopaminergic neurons in the pars compacta of substantia nigra. First parkinsonian motor symptoms occur after a loss of about 60 per cent of dopa-minergic nigral cells and after dopamine concentration in the posterior putamen has been reduced by about 80 per cent (Bernheimer JCS 1973; Deuschl 1999) [1, 2]. This presynap-tic nigrostriatal dopaminergic deficit in PD can be quantified by [^123^I]FP-CIT SPECT. FP-CIT SPECT measures the concentration of presynaptic striatal dopamine reuptake transporters (Booij Synapse 2001) [3]. Beyond the cerebral Lewy body type-degeneration, numerous studies found a Lewy body type-degeneration outside the brain in PD, too, primarily in the extracranial autonomous nervous system (Edwards Neurology 1992, Iwanaga Neurology 1999, Kaufmann Neurology 2004, Micieli NS 2003, Okada PI 2004, Wakabayashi EJ 1997) [4–9]. These studies show that PD does not affect only the brain. It is unclear, whether cerebral and extracranial Lewy body type-degeneration in PD are coupled or not.

[^123^I]MIBG scintigraphy measures the function of extracranial myocardial postganglionic sympathetic innervation. [^123^I]MIBG is a radioiodinated analogue of noradrenaline (norepinephrine). It is actively transported into noradrenaline granules of sympathetic nerve terminals by the noradrenaline transporter (Wieland JNM 1980) [10]. [^123^I]MIBG scintigra-phy reveals a high sensitivity concerning PD because it discloses an impaired myocardial postganglionic sympathetic innervation in PD (Braune CAR 2001, Hamada JNNP 2003, Yoshita NMC 1998) [11–13]. These data suggest that MIBG scintigraphy allows estimating the extent of Lewy body type-degeneration of the cardiac plexus, exemplarily of an extracranial structure.

Since there exists no information about the correlation between cerebral and extracranial Lewy body type-degeneration in PD, we applied FP-CIT SPECT and MIBG scintigraphy to address this point in PD. We used FP-CIT SPECT/MIBG scintigraphy to quantify nigrostriatal dopa-minergic/myocardial sympathetic degeneration. We looked for a correlation between cerebral nigrostriatal degeneration and extracranial myocardial sympathetic degeneration. Such a correlation would indicate that 1) cerebral nigrostriatal dopaminergic and extracranial myocardial sympathetic Lewy body type-degeneration are coupled and that 2) both degenerative processes result from identical or at least similar processes. Since stage, severity and duration of PD correlate significantly with reduction of nigrostriatal FP-CIT uptake (Benamer MD 2000) [14] and myocardial MIBG uptake (Hamada JNNP 2003) [12], we evaluated PD patients separately after Hoehn and Yahr stage to exclude a statistical bias due to the factors disease stage or severity.

## PATIENTS AND METHODS

### Patients and controls

#### Patients

The study involved 95 patients with idiopathic Parkin-son’s disease (PD; age 59 ± 11 years, mean ± SD, further details in Table 1). PD was diagnosed according to the criteria of the UK Parkinson’s Disease Society Brain Bank (Hughes JNNP 1992) [15]. Two different neurologists examined the patients. No patient was found to have a history of neuroleptic intake or other identifiable possible causes for secondary Parkinsonism. Cerebral MRI findings were normal in all patients, particularly regarding the absence of either white matter lesions or ventricular widening. Therapy with selegiline was discontinued at least 18 hours before FPCIT application due to possible interaction of its metabolites with striatal FP-CIT binding (Laruelle Synapse 1993) [16]. Further antiparkinsonian medication was continued during the time of scintigraphic examinations. Aware of the problem facing FP-CIT SPECT and MIBG scintigraphy in patients treated with antiparkinsonian drugs, the present data (Ahlskog MD 1999, Guttman Neurology 2001, Innis MD 1999, Laruelle Synapse 1993, Nurmi JCBFM 2000, Satoh JCN 1999, The Parkinson Study Group JAMA 2002) [16–22] did not allow a clear conclusion concerning the effect of dopaminergic drugs, neither on dopamine transporter binding, nor on MIBG distribution.

20 subjects (9 females, 11 males, age 37 – 74 years, 57 ± 11 years) underwent FP-CIT SPECT and MIBG scintigraphy because they were referred by the outpatient physician, who assumed a beginning PD. After PD had been excluded by clinical criteria and by the further clinical course, these subjects served as controls for the correlation between FP-CIT SPECT and MIBG scintigraphy. 11 of these 20 subjects were suffering from essential tremor, 2 from psychogenic Parkin-sonism, and 7 had no neurological or psychiatric disease. These controls were not significantly different from PD patients concerning age (p = 0.93; unpaired t-test). The norm values for FP-CIT SPECT and MIBG scintigraphy were *not* obtained from these controls but from other controls (see below).

In regard to the scintigraphic examinations, patients or controls with a clinical history of angina pectoris, arrhyth-mia, myocardial infarct, hypothyroidism, diabetes mellitus, cerebrovascular events, psychiatric disorders (except psy-chogenic Parkinsonism), malignancy, hypertension, phaeo-chromocytoma or any other disease, that might affect uptake of myocardial [^123^I]MIBG, were excluded. No subject was treated by tricyclic antidepressant drugs or other serotonin reuptake inhibitors, reserpine, clonidine, amphetamines, phenylpropanolamine, amiodarone or substances inducing any blockade of calcium channels. Written informed consent was obtained from all subjects prior to examination. The protocol was approved by the local ethics committee (Ethik-kommission der Ärztekammer des Saarlandes).

### Data Acquisition

#### [^123^I]FP-CIT SPECT

Cerebral SPECT imaging was performed with iodine 123 ioflupane (DaTscan^®^, Amersham Cygne, Eindhoven, The Netherlands). 4 hours following thyroid gland blocking and intravenous injection of the radioactive substance (110 - 185 MBq, specific activity: 580 - 1040 GBq/mg) cerebral SPECT images were obtained. A triple-head gamma camera (Siemens Multispect, MS 3) equipped with low-energy high-resolution collimators was used. Data was acquired in a 128 x 128 matrix covering 120 degrees per camera and head, 50 seconds per view. A total of 120 views was acquired. Energy discrimination was centered in 158 keV with a 15% window. Image filtering (Butterworth, Cutoff 0.5) was followed by attenuation correction (Chang, 0.11/cm). Registration and semiquantitative analysis were performed with a workstation (HERMES, Nuclear Diagnostics, Stockholm, Sweden) and a software named BRASS (Nuclear Diagnostics, Stockholm, Sweden), modified for this purpose. The caudate nucleus, putamen and occipital lobe binding of [^123^I]FP-CIT was assessed semiquantitatively by a regions of interest (ROI) technique and compared with a control group. The ROI’s included 524 pixels (according volume = 4.2 cm^3^) for each caudate nucleus, 689 pixels (according volume = 5.5 cm^3^) for each putamen, and 9008 pixels (according volume = 72 cm^3^) for both occipital cortices together. We regarded a small central area within each nucleus and did not consider the whole nucleus to avoid partial volume and resolution effects. We calculated the ratio *caudate nucleus/occipital lobe binding* and the ratio *putamen/occipital lobe binding.* Our norm values obtained from controls were previously described (Spiegel JNNT 2007) [23].

#### [^123^I]MIBG Scintigraphy

In a time period of 4 to 7 days preceding or following FP-CIT SPECT of the brain, MIBG examination of the heart took place: Following thyroid gland blocking with sodium, perchlorate intravenous injection of [^123^I]MIBG (Amersham, Amersham Buchler, Braunschweig, Germany) with a specific activity of 259 - 370 MBq/mg was given. The injected doses of MIBG ranged from 180 to 250 MBq. Planar whole-body images in anterior and posterior views were obtained 4 h after the injection using a dual-head large-field camera (Siemens Multispect, MS 2) equipped with low-energy high-resolution parallel-hole collimators. MIBG uptake of myocardium and mediastinum was quantified after drawing of a rectangular region of interest over the upper mediastinum and an irregularly configured region outlining the whole heart. Then the heart-to-mediastinum (H/M) count-ratio was calculated, which was defined as the average cts/pixel in the myocardium divided by that of the upper mediastinum. Our norm values obtained from controls were previously described (Spiegel MD 2005) [24]. Within one day after MIBG imaging, additional myocardial rest perfusion imaging was performed afterwards with ^99m^Tc-Sestamibi (MIBI, Cardio-lite^®^, Bristol Myers Squibh, USA, 500 MBq). This perfusion imaging was used to exclude a myocardial hypoperfusion that may influence MIBG imaging adversely. Patients with an impaired myocardial perfusion were excluded from further investigation. ther white matter lesions or ventricular widening. Therapy with selegiline was discontinued at least 18 hours before FP-CIT application due to possible interaction of its metabolites with striatal FP-CIT binding (Laruelle Synapse 1993) [16]. Further antiparkinsonian medication was continued during the time of scintigraphic examinations. Aware of the problem facing FP-CIT SPECT and MIBG scintigraphy in patients treated with antiparkinsonian drugs, the present data (Ahlskog MD 1999, Guttman Neurology 2001, Innis MD 1999, Laruelle Synapse 1993, Nurmi JCBFM 2000, Satoh JCN 1999, The Parkinson Study Group JAMA 2002) [16–22] did not allow a clear conclusion concerning the effect of do-paminergic drugs, neither on dopamine transporter binding, nor on MIBG distribution.

### Statistical Analysis

Correlations were calculated using the Pearson’s correlation coefficient in case of a normally distributed data and Spearman’s correlation coefficient in case of a not normally distributed data.

## RESULTS

### Patients

At all Hoehn and Yahr stages, caudate nucleus (CN) and putamen (PUT) FP-CIT uptake contralateral to the clinically more affected body side ( = contralateral uptake) were significantly smaller than CN/PUT FP-CIT uptake ipsilateral to the clinically more affected body side ( = ipsilateral uptake). Contralateral CN and/or PUT uptake was pathologically reduced in all patients.

**Fig. (1):**
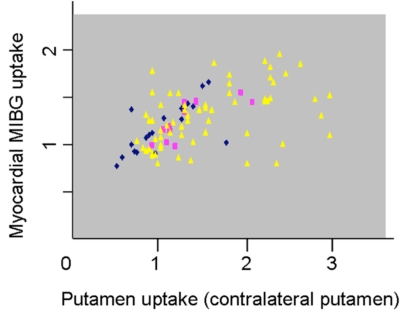
Correlation between putamen FP-CIT SPECT and MIBG scintigraphy. Each patient is represented by one symbol (patients with Hoehn and Yahr stage 1: yellow triangles, patients H&Y stage 2: red squares, patients H&Y stages 3 + 4: blue rhombs).

MIBG scintigraphy disclosed a pathologically reduced myocardial MIBG uptake in 88 of 95 patients. All patients with normal MIBG scintigraphy were at Hoehn and Yahr stage 1. At each Hoehn and Yahr stage separately, myocar-dial MIBG uptake correlated significantly with contralateral CN and PUT FP-CIT uptake (p < 0.05; Fig. 1). Furthermore, there was a significant correlation between MIBG uptake and ipsilateral CN and PUT FP-CIT uptake (p < 0.05; Pear-son’s correlation) at each Hoehn and Yahr stage. Multiple regression analysis showed that the correlation CN or PUT FP-CIT uptake versus myocardial MIBG uptake were not significantly influenced by the factors age, age at disease onset, disease duration or UPDRS (p > 0.05; multiple regression analysis).

### Controls

FP-CIT SPECT (n = 40 values due to bilateral measurement) and MIBG scintigraphy (n = 20) were normal in all 20 controls. For calculation of correlation between myocardial MIBG uptake and striatal FP-CIT uptake we considered the CN and PUT tracer uptake each of both sides: there was no significant correlation between MIBG uptake versus CN or PUT FP-CIT uptake.

## DISCUSSION

Idiopathic Parkinson’s disease (PD) is defined by the neuropathological finding of Lewy bodies (LB) primarily in the substantia nigra and other nuclear grays of the brain (Turner 1968) [25]. Based on the intracerebral formation of LB, Braak *et al.* (Braak CTR 2004) [26] defined six stages of PD. This sequence of cerebral LB degeneration is not random but results from the differing vulnerability and susceptibility of cerebral cell types to develop LB (Braak CTR 2004) [26]. Numerous studies found LB also in extracranial organs, especially in the peripheral autonomic nervous system: in the sympathetic system (intermediolateral nucleus of the thoracic cord and paravertebral sympathetic ganglia), the parasympathetic system (dorsal vagal and sacral parasympathetic nuclei; Iwanaga Neurology 1999, Kaufmann Neurology 2004, Wakabayashi and Takahashi EN 1997) [5, 6, 9], the adrenal medulla, and in the neural plexi innervating the gut, heart and pelvis (Edwards Neurology 1992, Micieli NS 2003, Okada PI 2004) [4, 7, 8]. These numerous histopa-thological changes at different parts of central and peripheral nervous system correlate with common clinical symptoms in PD such as constipation, orthostatic hypotension, detrusor hyperactivity or seborrhea (Deuschl 1999; Micieli NS 2003) [2, 7]. It is quite unclear whether there exists any relation between cranial and extracranial LB degeneration in PD. Furthermore the question arises whether cranial and extracranial LB degeneration are induced by the same pathome-chanisms and thus are coupled in PD.

In the present paper we studied whether there exists a correlation between the *cerebral* nigrostriatal dopaminergic function and the *extracranial* sympathetic myocardial function. We quantified *cerebral* nigrostriatal dopaminergic function with FP-CIT SPECT and *extracranial* sympathetic myocardial function with MIBG scintigraphy. We assumed that nigrostriatal dopaminergic function reflects the extent of nigral LB degeneration. Accordingly we measured the sympathetic myocardial function as a correlative for severity of myocardial sympathetic LB degeneration. Since stage, severity and duration of PD correlate significantly with reduction of nigrostriatal FP-CIT uptake (Benamer MD 2000) [14] and myocardial MIBG uptake (Hamada JNNP 2003) [12], we studied patients separately after Hoehn and Yahr stage to exclude a statistical bias due to the factors disease stage or severity.

We found a highly significant correlation between cerebral caudate nucleus (CN) and putamen (PUT) FP-CIT uptake versus extracranial myocardial MIBG uptake at all Hoehn and Yahr stages. Multiple regression analysis showed this significant correlation was not caused or influenced by the factors age, age at disease onset, disease duration or UPDRS. In the non-parkinsonian control subjects, there was no significant correlation between CN/PUT FP-CIT uptake versus extracranial myocardial MIBG uptake. This contradicts a physiological coupling of nigrostriatal dopaminergic and myocardial sympathetic function and proves that the close coupling between nigrostriatal degeneration and myo-cardial sympathetic degeneration in PD represents a pathological phenomenon.

## CONCLUSIONS

Our data indicate a strongly coupled degeneration of ni-grostriatal dopaminergic and myocardial sympathetic transmission at early as well as advanced stages of PD. We assume that at least *cerebral* nigrostriatal dopaminergic LB degeneration and *extracranial* myocardial sympathetic LB degeneration develop in a coupled manner. Obviously both processes are driven by identical or at least similar pathome-chanisms. Our results give strong evidence that the clinically and histopathologically manifest *cerebral* and *extracranial* degenerative changes in PD do not develop independently from each other *but* develop in a strongly coupled manner.

## Figures and Tables

**Table 1:** Demographic Data of Patients

	H&Y 1	H&Y 2	H&Y 3+4
N	63	12	20
Females	27	3	11
Males	36	9	9
Age (range)	37 – 78	40 – 71	42 – 76
Age	58 ± 10	56 ± 11	63 ± 8
UPDRS part III	11 ± 2	18 ± 3	30 ± 9

H&Y = Hoehn and Yahr stage (Hoehn Neurology 1967) [27]. No patient was at H&Y stage 5. Due to the case number of patients at H&Y stage 4 (n = 5) we combined the patients at H&Y stage 3 (n = 15) and H&Y stage 4 (n = 5) to one single group. N = number of patients. UPDRS part III = part III (motor part) of the Unified Parkinson’s disease rating scale (UPDRS, Fahn 1987) [28]. Antiparkinsonian medication had been stopped 12 hours before UPDRS scale was rated. Unless otherwise declared, all values are represented as mean ± SD.
